# Poor Status of Vitamin D: A Survey of Area With Lowest Sunlight Radiation in Sichuan, China

**DOI:** 10.3389/fendo.2021.626983

**Published:** 2021-02-24

**Authors:** Pianpian Fan, Qin Wang, Jing Li, Chunyan Lu, Yong Xu, Hongyi Cao, Xiaohua Xie, Xueyan Wu, Yanhong Guo, Ting Liu, Yan Chen, Shen Xu, Yuanyuan Huang, Qi Zhang, Decai Chen

**Affiliations:** ^1^ Department of Endocrinology, West China Hospital of Sichuan University, Chengdu, China; ^2^ Department of Endocrinology, The Affiliated Hospital of Southwest Medical University, Luzhou, China; ^3^ Department of Endocrinology, Chengdu Fifth People’s Hospital, Chengdu, China; ^4^ Department of Endocrinology, First People’s Hospital of Liangshan Yi Autonomous Prefecture, Xichang, China; ^5^ Department of Endocrinology, Guangyuan Central Hospital, Guangyuan, China; ^6^ Department of Endocrinology, Hospital of Chengdu Office of People’s Government of Tibetan autonomous Region, Chengdu, China; ^7^ Department of Endocrinology, Liangxiang Hospital, Beijing, China; ^8^ Department of Endocrinology, The First People’s Hospital of Neijiang, Neijiang, China

**Keywords:** vitamin D deficiency, lowest sunlight radiation, sunlight exposure, age, adult women

## Abstract

**Objective:**

Vitamin D plays an important role in bone and mineral metabolism. Ultraviolet B (UVB) is the primary determinant for vitamin D synthesis. However, population-based data of vitamin D status was sparse in areas with sunlight deprivation in China. This study aimed to assess serum 25-hydroxyvitamin D [25(OH)D] levels among adult women in Sichuan basin with the lowest sunlight radiation in China, and the associations with sunlight exposure and age.

**Methods:**

In the context of the same ethnicity, similar latitude and lifestyle in sunlight-limited basin and sunlight-abundant plateau, 1,057 women in basin and 337 in plateau aged 29–95 years were included in this study, from November 2012 to February 2013. Daily sunlight exposure duration of previous month was obtained using questionnaires. Serum 25(OH)D was measured by enzyme-linked immunosorbent assay.

**Results:**

The prevalence of vitamin D severe deficiency [25(OH)D <30 nmol/L] and deficiency [30 ≤ 25(OH)D <50 nmol/L] was significantly higher in basin than plateau (21.85% vs. 10.09%, and 59.32% vs. 40.36%; *P*<0.0001). Women from basin exhibited lower serum 25(OH)D levels than those from plateau (40.66 ± 15.62 vs. 52.54 ± 19.94 nmol/L, *P*<0.0001). In basin, women more than 50 years old had higher 25(OH)D than younger counterparts, and 25(OH)D level of these groups was not associated with sunlight exposure duration. While in plateau, women younger than 60 years old had higher 25(OH)D than the older women. Furthermore, for those younger groups, women with long sunlight exposure (≥3 h daily) had higher 25(OH)D concentration than those with short sunlight exposure (<3 h daily). Serum PTH was negatively associated with 25(OH)D in basin, but not in plateau.

**Conclusions:**

Alarmingly high prevalence of vitamin D deficiency was observed in women in sunlight-deprived basin in Sichuan. Only the vitamin D status of younger women from plateau with adequate solar radiation could benefit from sunlight exposure. Vitamin D supplementation and vitamin D-fortified food should be encouraged to improve vitamin D status for women living in sunlight-limited areas, or with old age.

## Introduction

Vitamin D plays an important role in bone and mineral metabolism, skin function, immune regulation, and vascular health. However, more than one billion children and adults worldwide have been estimated to have vitamin D deficiency or insufficiency (1). The source of vitamin D in human are cutaneous production, vitamin D-fortified diet and vitamin D supplements, the former accounts for at least 80% (2). Sufficient ultraviolet B (UVB) radiation initiates the conversion of 7-dehydrocholesterol (7DHC) into previtamin D_3_ in skin (3). As an important external impact factor for vitamin D synthesis, UVB radiation is determined by season, latitude, and weather conditions. Additionally, age, skin pigmentation, unprotected skin area exposed, and sunlight exposure duration have been identified as internal factors for vitamin D status (3).

China is a large country with heterogeneous geographic conditions, sun radiation intensity, dietary habits and clothing styles, resulting in various prevalence of vitamin D deficiency in different areas. A study conducted in 2,173 adults aged 18–65 years demonstrated that vitamin D deficiency [25(OH)D <50 nmol/L] was observed in 39.6–42.1% of residents in coastal cities and 60.6–73.5% in non-coastal cities (4). Zhen et al. screened 10,038 adults aged 40–75 years old in northwestern China, and found that 75.2% of them had vitamin D deficiency (<50 nmol/L), and women and old age were risk factors (5). One recent study in Tianjin Province showed that prevalence of vitamin D deficiency (<50 nmol/L) was 61.31% in women (6). However, many studies were conducted in summer or autumn. Few population-based studies were performed in winter, when people may not be able to achieve adequate vitamin D through sunlight exposure.

Sichuan Province is located in southwestern China (26°03′–34°19′ N, 97°21′–108°31′ E), with a population of 81 million and diverse terrain consisting of both basin and plateau. In basin, a subtropical monsoon climate results in it one of area with the shortest sunshine duration and weakest ultraviolet radiation intensity in China (7). More specifically, the annual duration of sunshine in this region is approximately 600 h (7); additionally, this region has low UVB radiation (less than 4,000 MJ/m^2^ annually) and UV index (a measure of UV intensity weighted for erythema response, only 2–6 in winter) (7–9). However, only 400 kilometers away from basin, the Sichuan plateau has long sunshine duration (>2200 h annually), high UVB radiation (5,100–6,000 MJ/m^2^ annually), and high UV index (with 5–8 even in winter) (7–9). Therefore, in the context of the same ethnicity, similar latitude and lifestyle in basin and plateau, Sichuan Province offered a unique model for investigating the effect of sunlight exposure on vitamin D status of residents. While no similar studies have been previously carried out in this area.

This population-based study aimed to investigate serum 25(OH)D level of adult women in sunshine-deficient basin during winter, and the effects of sunlight exposure and age on vitamin D status.

## Participants and Methods

### Study Design and Participants

The data of this study from a cross-sectional population-based survey conducted in Sichuan Province of China, from November 2012 to February 2013 (winter weather). It was designed to evaluate the vitamin D status and bone health of adult women in Sichuan Province. Briefly, four cities were selected, Guangyuan (32°26′ N), Luzhou (28°54′ N), Chengdu (30°40′ N) and Xichang (27°55′ N) located in north, south, east and farther south of Sichuan, respectively. The first three cities are characterized by basin terrain, weak solar radiation and short sunshine duration, while the later one is featured with highland plateau terrain, intense solar radiation and long sunshine duration (**Supplemental Table S1**). These four cities were selected to represent geographically distinct areas in Sichuan Province, with comparable latitudes and similar lifestyles. Cluster sampling was used for study population recruitment. Two urban communities and 1–2 rural communities were randomly selected in each city mentioned above. Women who had been living in their current community for at least 10 years were invited to participate in this study by advertising. Participants were randomly selected within strata based on age. The number of women recruited was about 1:1 in these four cities, as well as in urban and rural community of each city. This study was approved by the Institutional Review Board of the West China Hospital of Sichuan University. Signed informed consents were obtained from all participants at study enrollment.

**Figure 1:**
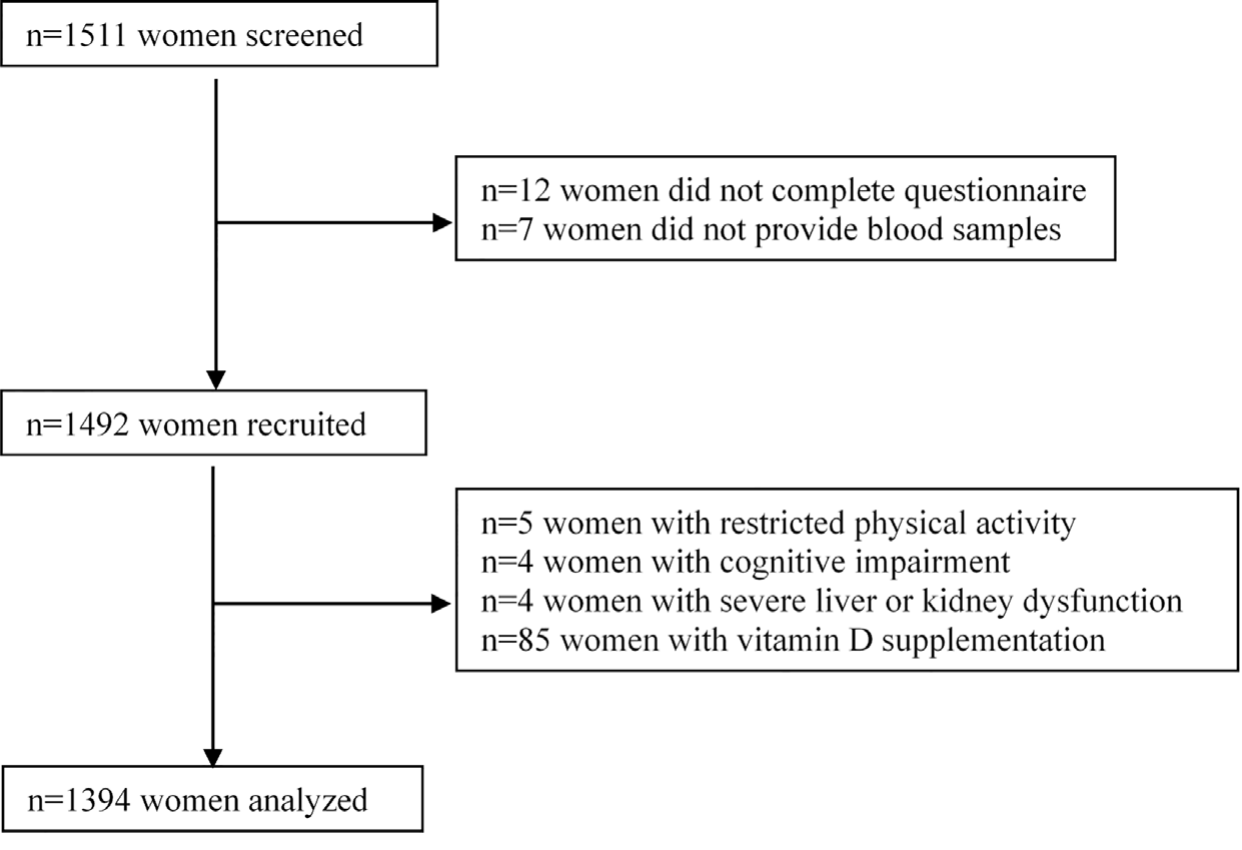
Flow chart for participants recruited.

This study screened 1,511 women aged 29–95 years. Of these, 1,499 participants completed questionnaires, of whom 1,492 provided blood samples. Women with restricted physical activity (n=5), cognitive impairment (n=4), or severe liver or kidney dysfunction (n=4) were excluded. To better understand the effects of sunlight exposure on 25(OH)D level, participants who took vitamin D supplements in the past month (n=85) were excluded from data analysis. A total of 1,394 participants were included in the final analysis (**Figure 1**).

### Data Collection

In this study, questionnaires were administered by trained staff to collect information according to a standard protocol at the four cities. Vitamin D supplement that participants took in the past month included native vitamin D (vitamin D_2_, vitamin D_3_) and active vitamin D (calcifediol, calcitriol). Calcium supplement took in the previous month included calcium carbonate and calcium gluconate. Dietary calcium intake was evaluated *via* food frequency questionnaire. Chinese Food Composition Tables (10) was referenced to transform daily food consumptions into dietary calcium intakes, which were the sum of calcium in each food items. Sunlight exposure duration was assessed by asking “How long did you spend outdoors per day on average during the previous month?”. The time spent outdoors was further categorized into “long sunlight exposure” (LSE, ≥3 h daily) and “short sunlight exposure” (SSE, <3 h daily). The sun protection habits included using sunscreen and umbrella, and its frequency was classified as never, occasionally (including “rarely” and “sometimes”) and frequently (“often” and “always”). Height and weight were measured to calculate body mass index (BMI; in kg/m^2^). Considering the distinct terrain, sunlight radiation and sunshine duration in basin and plateau, based on the current residential city of participants, women from Guangyuan, Luzhou or Chengdu were categorized into basin group, and those from Xichang into plateau group. According to their residential community, the participants were classified into rural or urban area group.

### Measurements for Serum 25(OH)D, Parathyroid Hormone, Calcium, and Phosphate

Fasting blood samples were collected, rested for 30 min and then centrifuged. Aliquotes of serum samples were stored at -80°C until analysis. As a suitable rapid automated assay for 25(OH)D analysis for use in the clinical diagnostic laboratory (11), enzyme-linked immunosorbent assay (ELISA) was used to measure serum 25(OH)D (Immunodiagnostic Systems, IDS Ltd., London, UK). Serum parathyroid hormone (PTH) was measured with electrochemiluminescence immunoassays on the Roche cobas e601 analyzer. Serum calcium and phosphate were measured on the Roche cobas 8000 analyzer. All the measurements were conducted in the clinical laboratory of the West China Hospital; the lab is certified by the China National Accreditation Board. QA/QC procedures were performed for all analyses in accordance with the system’s instructions. The intra- and inter- assay coefficients of variation (CVs) were 5.3%–6.7% and 4.6%–8.7% for 25(OH)D, 1.8%–3.2% and 7.0%–7.7% for PTH. The inter assay CVs for calcium and phosphate were 1.8% and 1.5%, respectively.

In our study, severe vitamin D deficiency, vitamin D deficiency, insufficiency, and sufficiency were predefined as serum 25(OH)D concentrations <30 nmol/L; ≥30 but <50 nmol/L; ≥50 but <75 nmol/L; and ≥75 nmol/L, respectively, according to recommendations by the Endocrine Society in 2011 (12).

### Statistical Methods

Continuous variables were expressed as the mean ± standard deviation. Categorical data were shown as percent. For comparison of continuous variables, independent sample *t* test was used. Chi-square was performed for categorical data. Linear regressions were used for the association with 25(OH)D. To examine the associations with age, sunlight exposure (<3 h daily, ≥3 h daily) and region (basin, plateau), generalized linear models were adjusted for BMI, education level, exposed site, sun protection, and living in rural/urban area. To examine the associations with serum level of PTH, calcium and phosphate, calcium supplement intake, daily dietary calcium intake and menopausal status were additionally adjusted. A two-tailed *P*<0.025 was defined as statistically significant. All analyses were performed using the SAS 9.4 software (SAS Institute, Inc, Cary, NC, USA).

## Results

### Characteristics of Participants in Basin and Plateau

Characteristics of 1,394 women in basin and plateau were presented in **Table 1**. The average age was 58.18 ± 13.85 and 59.64 ± 14.90 years in basin and plateau. Lower percent of postmenopausal women was observed in basin than in plateau (63.39% vs. 69.44%, *P*=0.043). Compared with women in plateau, the women in basin had higher BMI (24.23 ± 3.37 vs. 23.66 ± 3.66 kg/m^2^, *P*=0.008), lower percent with college degree or above (15.56% vs. 32.59%, *P*<0.0001), and similar percent from urban community. No significant difference in average sunlight exposure duration was observed between women from these two areas. Similar percent of individuals took sun protection in basin and in plateau, while women in plateau had higher percentile of hands exposure (99.11% vs. 93.12%, *P*<0.0001). No significant difference in calcium supplement or dietary calcium intake was observed between basin and plateau. Women in basin had lower serum calcium but higher phosphate than women in plateau.

**Table 1:** Characteristics of participants in basin and plateau.

Variable	Basin (n = 1057)	Plateau (n = 337)	*P*-value
Age (years)	58.18 ± 13.85	59.64 ± 14.90	0.098
Age group [% (n)]			0.193
29–39	9.87 (104)	11.28 (38)	
40–49	22.87 (241)	18.99 (64)	
50–59	20.97 (221)	18.99 (64)	
60–69	21.16 (223)	20.18 (68)	
70–95	25.14 (265)	30.56 (103)	
Post-menopause [% (n)]	63.39 (670)	69.44 (234)	**0.043**
BMI (kg/m^2^)	24.23 ± 3.37	23.66 ± 3.66	**0.008**
Education [% (n)]			**<0.0001**
High school or lower	84.44 (803)	67.41 (151)	
College/university	15.56 (148)	32.59 (73)	
Residential community [% (n)]			0.318
Urban	50.05 (529)	46.88 (158)	
Rural	49.95 (528)	53.12 (179)	
Sunlight exposure (hours, daily)			
<3	60.36 (638)	56.97 (192)	0.279
≥3	39.64 (419)	43.03 (145)	
Exposed site [% (n)]^#^			
Face	97.36 (665)	97.63 (329)	1.000
Hands	93.12 (636)	99.11 (334)	**<0.0001**
Arms	8.05 (55)	6.23 (21)	0.314
Legs	3.23 (22)	2.67 (9)	0.702
Sun protection [% (n)]^#^			0.798
Never	68.68 (467)	72.70 (245)	
Occasionally	24.85 (169)	15.73 (53)	
Frequently	6.47 (44)	11.57 (39)	
Calcium supplement [% (n)]	43.14 (456)	42.73 (144)	0.894
Daily dietary calcium intake (mg)	435.8 ± 534.3	384.4 ± 328.5	0.058
25(OH)D (nmol/L)	40.66 ± 15.62	52.54 ± 19.94	**<0.0001**
PTH (pg/mL)	64.86 ± 48.47	68.88 ± 36.86	0.108
Calcium (mmol/L)	2.36 ± 0.10	2.40 ± 0.10	**<0.0001**
Phosphate (mmol/L)	1.14 ± 0.17	1.01 ± 0.18	**<0.0001**

BMI, body mass index; 25(OH)D, 25-hydroxyvitamin D; PTH, parathyroid hormone. ^#^exposed site and sun protection were able to be abstracted from questionnaire of 686 participants. Figures in bold means p value < 0.05.

### Vitamin D Status in Basin and Plateau

**Figure 2:**
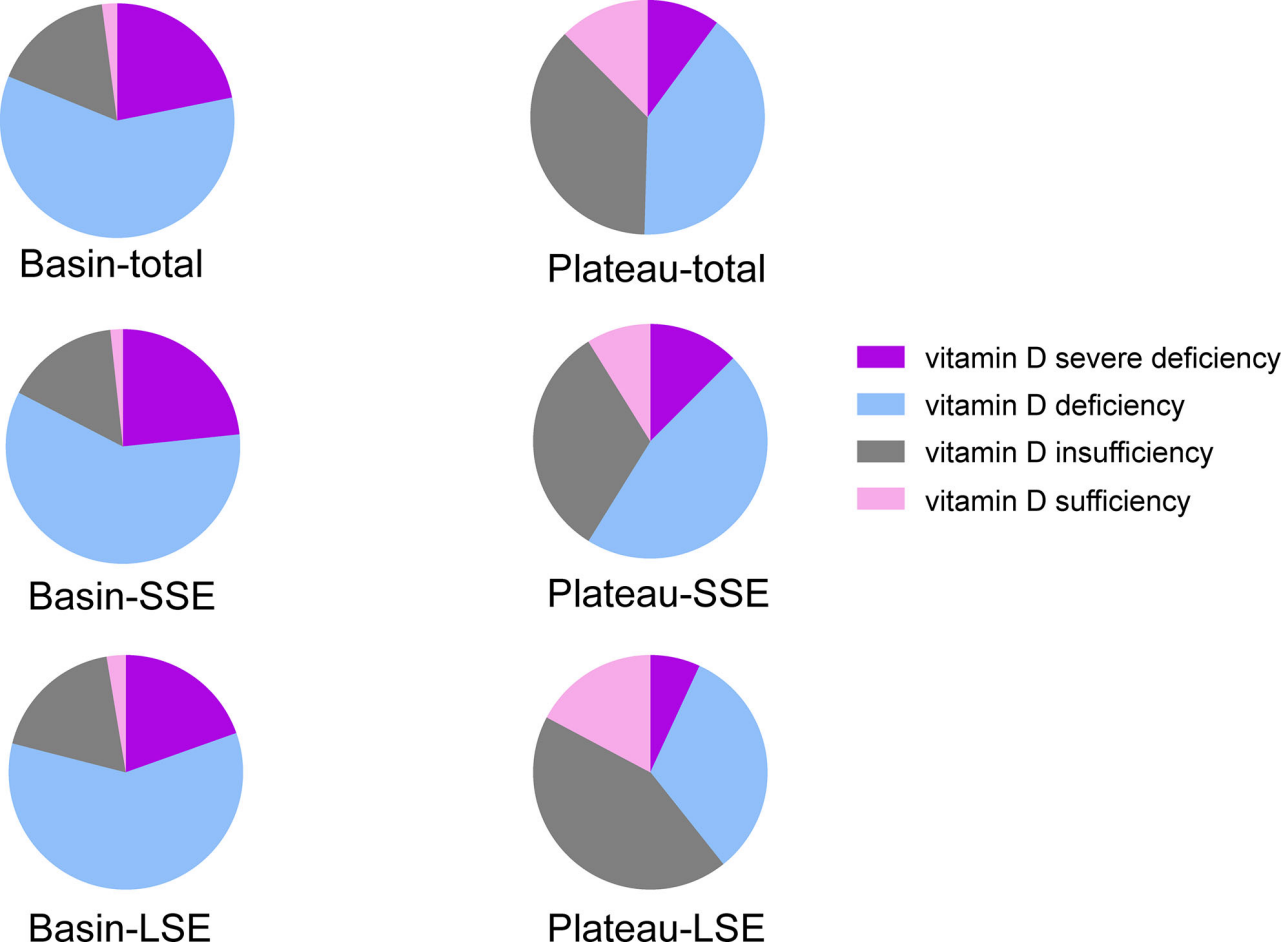
Distribution of 25(OH)D categorized by region and sunlight exposure. Total, total participants in this region; SSE, short sunlight exposure, <3 hours daily; LSE, long sunlight exposure, ≥3 hours daily.

For the three cities in basin (Guangyuan, Luzhou and Chengdu), the serum 25(OH)D level in Luzhou was lower than that in Chengdu (*P*=0.028, **Supplementary Table S2**). Women in basin exhibited significantly lower serum 25(OH)D levels than those from plateau (40.66 ± 15.62 vs. 52.54 ± 19.94 nmol/L, *P*<0.0001) (**Table 2**). **Table 2** and **Figure 2** presented the 25(OH)D category by region and sunlight exposure. The prevalence of vitamin D severe deficiency, deficiency, insufficiency, and sufficiency were 21.85%, 59.32%, 16.75%, and 2.08% in women from basin, compared with 10.09%, 40.36%, 37.09%, and 12.46% in women from plateau, respectively (*P*<0.0001). In plateau, women with long sunlight exposure had higher proportion of vitamin D insufficiency and sufficiency than those with short sunlight exposure (*P*=0.0003), while in basin, no significant difference in prevalence of vitamin D deficiency between women with long or short sunlight exposure.

**Table 2:** Distribution of 25(OH)D categorized by region and sunlight exposure.

25(OH)D	Basin			Plateau		
	SSE (n = 638)	LSE (n = 419)	Total (n = 1057)	SSE (n = 192)	LSE (n = 145)	Total (n = 337)
Mean ± SD (nmol/L)	39.81 ± 15.78	41.97 ± 15.30	40.66 ± 15.62	48.73 ± 17.98^a^	57.58 ± 21.31^ab^	52.54 ± 19.94^a^
25(OH)D group [% (n)]						
severe deficiency	23.35 (149)	19.57 (82)	21.85 (231)	12.50 (24)	6.90 (10)	10.09 (34)
deficiency	59.25 (378)	59.43 (249)	59.32 (627)	46.35 (89)	32.41 (47)	40.36 (136)
insufficiency	15.67 (100)	18.38 (77)	16.75 (177)	32.29 (62)	43.45 (63)	37.09 (125)
sufficiency	1.72 (11)	2.63 (11)	2.08 (22)	8.85 (17)	17.24 (25)	12.46 (42)

25(OH)D, 25-hydroxyvitamin D; SSE, short sunlight exposure, <3 hours daily; LSE, long sunlight exposure; ≥3 hours daily.

^a^P < 0.0001 compared with the same sunlight exposure duration group in basin; ^b^P < 0.01 compared with short sunlight exposure duration group in plateau. Models were adjusted for BMI, education level, exposed site, sun protection, and living in rural/urban area.

### The Association Between 25(OH)D Levels With Sunlight Exposure and Age

**Figure 3:**
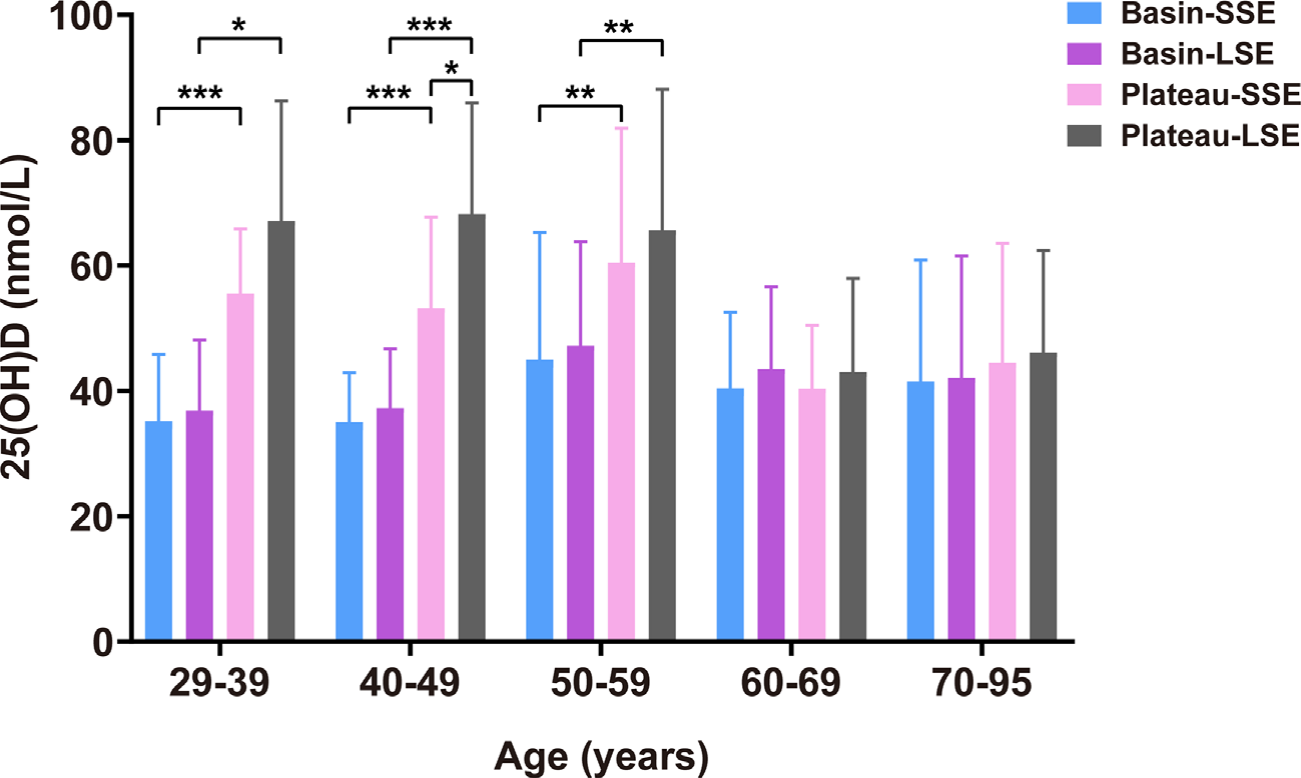
Serum 25(OH)D levels stratified by region, age and sunlight exposure duration. SSE, short sunlight exposure, <3 hours daily; LSE, long sunlight exposure, ≥3 hours daily. Models were adjusted for BMI, education level, exposed site, sun protection, and living in rural/urban area. *P < 0.05; **P < 0.001; ***P < 0.0001.

Serum 25(OH)D levels of participants from basin and plateau were stratified by sunlight exposure duration and age (**Table 3**, **Figure 3**). In basin, women more than 50 years old had higher 25(OH)D than those younger counterparts in both short and long sunlight exposure group, but not significant in the latter. At each age group, women with short sunlight exposure had similar 25(OH)D concentration with those exposed to long sunlight exposure. In plateau, women younger than 60 years old had higher 25(OH)D than the older women, in both short and long sunlight exposure group. For those younger groups, women with long sunlight exposure had higher 25(OH)D concentration than those with short sunlight exposure, though significant difference was only observed in age 40–49 years group.

**Table 3:** Serum 25(OH)D concentration (mean±SD) by age and sunlight exposure in basin and plateau.

Age group(years)	Basin				Plateau			
	SSE	β (95%CI)	LSE	β (95%CI)	SSE	β (95%CI)	LSE	β (95%CI)
29–39	35.19 ± 10.66	Ref	36.84 ± 11.29	Ref	55.54 ± 10.32	Ref	67.12 ± 19.13	Ref
40–49	35.04 ± 7.86	0.10 (-5.51,5.71)	37.29 ± 9.44	-5.59 (-15.41,4.24)	53.20 ± 14.53	-4.07 (-14.01,5.88)	68.21 ± 17.77	5.40 (-9.17,19.97)
50–59	44.98 ± 20.27	7.66 (1.62,13.70)*	47.18 ± 16.58	3.36 (-6.74,13.45)	60.47 ± 21.45	2.41 (-8.27,13.08)	65.63 ± 22.48	6.23 (-10.75,23.21)
60–69	40.39 ± 12.16	6.39 (-0.19,12.97)	43.47 ± 13.13	5.16 (-4.88,15.21)	40.37 ± 10.08	-16.10 (-27.83,-4.37)**	42.99 ± 14.96	-19.76 (-35.81,-3.71)*
70–95	41.46 ± 19.41	7.73 (1.58,13.88)*	42.05 ± 19.48	0.58 (-9.72,10.88)	44.48 ± 19.07	-13.47 (-24.35,-2.58)*	46.12 ± 16.27	-2.94 (-25.32,19.44)
*P* trend	–	**0.002**	–	0.062	–	**0.007**	–	**0.025**

SSE, short sunlight exposure, <3 hours daily; LSE, long sunlight exposure, ≥3 hours daily.

Models were adjusted for BMI, education level, exposed site, sun protection, and living in rural/urban area.

*P < 0.05; **P < 0.01. Figures in bold means p value < 0.05.

For women younger than 60 years old, women in basin had significantly lower 25(OH)D than those in plateau, in both short sunlight exposure and long sunlight exposure (**Figure 3**). However, for women older than 60 years, they had similar 25(OH)D concentration, no matter exposed to short or long sunlight, or living in basin or in plateau (**Figure 3**).

### The Association Between 25(OH)D Levels With Serum PTH, Calcium, and Phosphate

In basin, serum PTH was negatively associated with 25(OH)D (**Table 4**). Compared with that in women with vitamin D severe deficiency, PTH was 12.08 (95%CI: 4.01, 20.14) pg/mL lower in women with vitamin D deficiency, and 27.41 (95%CI: 0.15, 54.66) pg/mL lower in women with vitamin D sufficiency. In plateau, neither significant association between 25(OH)D with PTH, nor with calcium or phosphate was observed.

**Table 4:** Association between serum 25(OH)D with PTH, calcium, and phosphate in basin and plateau.

25(OH)D group	PTH		Calcium		Phosphate	
	Mean ± SD (pg/mL)	β (95%CI)	Mean ± SD (mmol/L)	β (95%CI)	Mean ± SD (mmol/L)	β (95%CI)
**Basin**						
severe deficiency	72.19 ± 54.57	Ref	2.35 ± 0.10	Ref	1.14 ± 0.21	Ref
deficiency	61.02 ± 37.32	-12.08 (-20.14,-4.01)**	2.36 ± 0.11	0.01 (-0.01,0.03)	1.14 ± 0.17	0.01 (-0.02,0.04)
insufficiency	62.23 ± 41.18	-10.41 (-21.68,0.85)	2.36 ± 0.10	0.01 (-0.02,0.03)	1.16 ± 0.13	0.002 (-0.05,0.05)
sufficiency	42.75 ± 30.53	-27.41 (-54.66,-0.15)*	2.41 ± 0.06	0.05 (-0.01,0.12)	1.12 ± 0.16	-0.03 (-0.14,0.09)
* P* trend	–	**0.008**	–	0.266	–	0.992
**Plateau**						
severe deficiency	82.26 ± 36.24	Ref	2.40 ± 0.10	Ref	1.01 ± 0.18	Ref
deficiency	72.77 ± 41.06	-10.09 (-26.53,6.34)	2.39 ± 0.09	0.003 (-0.04,0.05)	1.02 ± 0.19	0.04 (-0.04,0.12)
insufficiency	61.68 ± 32.50	-10.99 (-27.94,5.96)	2.40 ± 0.09	0.01 (-0.04,0.05)	1.01 ± 0.17	0.03 (-0.06,0.11)
sufficiency	66.85 ± 31.10	-11.83 (-31.30,7.63)	2.40 ± 0.12	-0.01 (-0.07,0.04)	1.00 ± 0.20	0.02 (-0.08,0.11)
* P* trend	–	0.303	–	0.703	–	0.937

25(OH)D: 25-hydroxyvitamin D; PTH, parathyroid hormone.

Models were adjusted for BMI, education level, exposed site, sun protection, living in rural/urban area, calcium supplement intake, daily dietary calcium intake and menopausal status.

*P < 0.05; **P < 0.01. Figures in bold means p value < 0.05.

## Discussion

This cross-sectional population-based study showed an alarmingly high prevalence of vitamin D deficiency in adult women during winter, in sunlight-deprived basin of Sichuan, China, and no significant association was observed between 25(OH)D with age and sunlight exposure duration. However, in sun-abundant plateau, women with young age and long sunlight exposure had higher 25(OH)D level than their counterparts. Serum PTH was negatively associated with 25(OH)D in basin, but not in plateau.

Our study demonstrated alarmingly poor vitamin D status in Sichuan Province, especially in the sunlight-deprived basin, with 81.17% of women found to be vitamin D deficient. It was consistent with a recent study conducted in Sichuan Province (13). The prevalence of vitamin D deficiency in our study was markedly higher than the nationwide average with a prevalence of 66.3% (4) and those of most provinces in China (14, 15). To the best of our knowledge, the highest prevalence of vitamin D deficiency [25(OH)D <50 nmol/L] in Chinese women was 82.5% in Lanzhou (16), an area with a higher latitude than that of Sichuan Province.

The vitamin D status in the Sichuan basin is considerably poor when compared worldwide. The National Health and Nutrition Examination Survey (NHANES) showed that the prevalence of vitamin D deficiency [25(OH)D <50nmol/L] in Hispanic, non-Hispanic white, non-Hispanic black, and non-Hispanic Asian women were 32.2%, 13.9%, 53.3%, and 36.7%, respectively (17). While both of the black and Hispanic women had darker skin pigmentation than women in our study. Not only the skin color and phototype could influence the 25(OH)D serum levels but other genetic factors. For example, American Indians appear to have a decrease in the production of vitamin D by the dermis and a possible increase in 25(OH)D_3_-24-hydroxylase (24OHase) with increased degradation of 25(OH)D. In comparison to Caucasians, these Indians have lower serum levels of 25(OH)D (18–20). The German Health Interview and Examination Survey for Adults (DEGS1) showed that 61.5% of adult women had vitamin D deficiency (<50 nmol/L) (21). The Korea National Health and Nutrition Examination Surveys (KNHANES) conducted from 2008 to 2014 showed that vitamin D deficiency (<50 nmol/L) was found in 76.7% of females in overall population (22). The prevalence of vitamin D deficiency in Japanese women was 54.6% (23).

Our study showed that the prevalence of vitamin D deficiency in basin was significantly higher than that in plateau, which was largely contributed by the different sun radiation intensity. Basin has a short sunlight duration (approximately 600 h annually) and low UVB radiation (less than 4,000 MJ/m^2^ annually), while plateau has long sunshine duration (>2200 h annually) and high UVB radiation (5,100–6,000 MJ/m^2^ annually). The UV index for the months November to February was 2–6 in basin but 5–8 in plateau, for June to August 10–12 in basin and 12–14 in plateau (7–9). The 25(OH)D levels were expected to be higher in summer than those in winter, in agreement with previous studies (13). Our study was conducted in winter season, providing better understanding for the lowest level of 25(OH)D in the all year around. One study conducted in participants aged 7–18 years showed that even sufficient vitamin D level in summer did not provide assurance of vitamin D sufficiency in winter (24). Longitudinal multi-center survey in basin and plateau is needed to evaluate the effect of solar radiation in summer on 25(OH)D level in winter. During the winter at latitudes above ~35°, there is almost no previtamin D_3_ production in the skin (25). We also observed that no significant association between 25(OH)D levels and sunlight exposure duration in basin. It was consistent with previous studies (26, 27).

To be noticed, a nearly twice difference in UVB intensity between plateau and basin only led to rather minor difference in mean serum 25(OH)D levels. It may be explained by that the production of cholecalciferol by sunlight exposure is self-limited as excessive sunlight exposure results in degradation of previtamin D_3_ and vitamin D (3, 28). In addition, during prolonged sunlight exposure, the maximized accumulation of previtamin D_3_ is less than 15% of the original 7-DHC content (3). In addition, study showed that 10 times increase of the serum vitamin D_3_ levels could only double the serum 25(OH)D levels (29).

Long sunlight exposure duration increased 25(OH)D level in women in plateau. It agreed with previous studies (30). The primary source of vitamin D for most human is sunlight exposure between approximately 9:00 and 15:00 h (local solar time) during the spring, summer and autumn seasons (31). The recommended way to increase vitamin D levels is by short-time and regular exposures, large amount of unprotected skin to sunlight (32). For example, 20 min of sunlight exposure three times per week could ensure adequate vitamin D levels in Caucasians. However, darker skinned peoples, old individuals, and those living at high latitudes may not achieve adequate vitamin D through sunlight exposure (33). For such people, exogenous supplementation should be considered. Due to the limited availability of vitamin D oral supplements and vitamin D-fortified food in many countries worldwide, the recommendations for adults suggested 1,000 to 2,000 IU/day of cholecalciferol (33). Alternative regimens using high dose intermittent therapy were also commonly used, for example, ergocalciferol 50000 IU weekly or monthly (33). Exposure to lamps that produce UVB radiation was also an alternative to produce vitamin D_3_ for some individuals (25).

Age is an important factor influencing vitamin D status, as our study showed that older age was associated with lower vitamin D levels in plateau, which was consistent with other epidemiological studies (5, 6, 34). Previous study has suggested that when exposed to equal amounts of sunlight, a 70-year-old man could only synthesize 25% of the vitamin D produced by a 20-year-old man (35). Aging may affect 7DHC levels in epidermis cells, then resulted in reduced formation of previtamin D_3_ (36). This reduction also seems to be related to wearing more clothing, reducing physical activity and less time spent outdoors (37).

We also noticed that individuals from basin aged more than 50 years had higher vitamin D levels compared to those aged 29–49 years in basin. It agreed with several previous studies. The KNHANES observed poorer vitamin D status in participants aged 20 to 49 years compared to those aged over 50 years (38). Considering the fact that the fluctuation of blood 25(OH)D levels through the year was similar with daylight and global solar radiation but delayed by 2 months (39), and 25(OH)D has a half-life of approximately 2 to 3 weeks (40). It assumed that the older women were less likely to take sun protection in summer and autumn, therefore, more vitamin D was synthesized and stored, and then utilized in winter (38, 41).

We observed that PTH was negatively associated with 25(OH)D in basin. It was consistent with previous studies (4, 42). PTH and vitamin D, two important regulators of bone and mineral metabolism, are essential for the maintenance of calcium and phosphate homeostasis as well as bone health. PTH and vitamin D form a tightly controlled feedback loop. PTH stimulates vitamin D synthesis in kidney while vitamin D suppresses PTH secretion. However, this negative feedback was not observed in plateau in our study. The ideal 25(OH)D concentration are generally not associated with any marginal increase in serum PTH (43). Another study also showed increases in the cut-off value for serum 25(OH)D did not change the range of serum PTH (18). One study demonstrated that circulating PTH concentration was maximally suppressed when the serum 25(OH)D was 50 nmol/L in women (44). The average level (mean ± SD) of 25(OH)D was 52.54 ± 19.94 nmol/L in our study, it may suggest that the 25(OH)D level in plateau have exceed the threshold of feedback. In addition, calcium intake also interacts with the relationship between 25(OH)D and PTH (45).

This study had some strengths, in the context of similar latitude and the same ethnicity in basin and plateau, Sichuan Province offered a unique model for investigating the effect of sunlight exposure on vitamin D status of residents. Large sample size provided robust estimates of vitamin D status. This study also had several limitations. We did not measure the UVB intensity, and used self-reported daily duration of outdoor activity as proxy for sunlight exposure duration. The cross-sectional study design precluded the drawing of cause-effect conclusions. This study had no external quality control assessment from International Vitamin D External Quality Assessment Scheme (DEQAS) to the 25(OH)D measurement, though ELISA for measuring 25(OH)D had a good agreement with liquid chromatography–tandem mass spectrometry (LC–MS/MS) (11), potentially the most accurate and precise method in research studies (46). In addition, nutritional intake of vitamin D was not considered in this study. Low availability of vitamin D-fortified foods (47), and low consumption of vitamin D-containing food in Sichuan (only 18.44% of women consumed milk more than 250 g daily in our study) suggested low nutritional intake of vitamin D.

## Conclusions

This population-based study demonstrated vitamin D deficiency is highly prevalent among adult women in Sichuan basin during winter. Increasing sunlight exposure was only beneficial to the vitamin D status of younger individuals in areas with abundant sun radiation. Increasing the availability of vitamin D supplements and vitamin D-fortified food should be the main strategies to improve vitamin D status in sunlight-limited areas.

## Data Availability Statement

The raw data supporting the conclusions of this article will be made available by the authors, without undue reservation.

## Ethics Statement

The studies involving human participants were reviewed and approved by Institutional Review Board of the West China Hospital of Sichuan University. The participants provided their written informed consent to participate in this study.

## Author Contributions 

QW and DC conceived and designed the study. QW, DC, YX, HC, XX, and XW coordinated and conducted the survey. YG, TL, and YC collected data. PF, QW, SX, YH, and QZ analyzed the data. PF, QW, JL, and DC interpreted the data. PF, QW, JL, and DC drafted the manuscript. PF, QW, JL, CL, and DC revised the manuscript. DC had primary responsibility for final content. All authors contributed to the article and approved the submitted version.

## Funding

These data were from the Vitamin D Status and its Correlation to Bone Density, Bone Turnover Markers and Muscle Strength in China (IISP-40407) project, which was founded by Merck Sharp and Dohme. However, Merck Sharp and Dohme had no role in the study design and conduct; data collection, analysis and interpretation; or manuscript preparation and approval.

## Conflict of Interest

The authors declare that the research was conducted in the absence of any commercial or financial relationships that could be construed as a potential conflict of interest.
