# Supplementary material for: Poor Status of Vitamin D: A Survey of Area With Lowest Sunlight Radiation in Sichuan, China
Source: Front Endocrinol (Lausanne). 2021 Feb 24;12:626983. doi: 10.3389/fendo.2021.626983 (PMC7959743; doi:10.3389/fendo.2021.626983)
Supplement: Supplementary file 1 [file Table_1.docx]

**Supplemental table S1**. Geography and solar radiation of Guangyuan, Luzhou, Chengdu and Xichang

| **City** | **Terrain** | **Average altitude (meters)** | **Average longitude** | **Average latitude** | **Average sunlight during investigation (kWh/m^2^/day) ^a^** |
| --- | --- | --- | --- | --- | --- |
| Guangyuan | Basin | 487 | 105°51' E | 32°26' N | 2.22 |
| Luzhou | Basin | 337 | 105°23' E | 28°54' N | 1.96 |
| Chengdu | Basin | 505.9 | 104°03' E | 30°40' N | 2.34 |
| Xichang | Plateau | 1590.7 | 102°17' E | 27°55' N | 4.24 |

^a^ Average surface meteorology and solar energy (SSE) data from atmospheric science data center of NASA (https://power.larc.nasa.gov/data-access-viewer/)

**Supplemental table S2**. Serum 25(OH)D concentrations of adult women in basin and plateau

| **Region** | **N** | **Mean**±**SD** |  | **Percentiles** | | | | | | | **Range (nmol/L)** |
| --- | --- | --- | --- | --- | --- | --- | --- | --- | --- | --- | --- |
|  |  |  | **2.5^th^** | | **5^th^** | **25^th^** | **50^th^** | **75^th^** | **95^th^** | **97.5^th^** |  |
| Total | 1394 | 43.53±17.51 | 21.38 | | 23.18 | 32.31 | 40.21 | 50.51 | 74.05 | 86.26 | 12.60-206.72 |
| Basin | 1057 | 40.66±15.62 | 21.38 | | 23.01 | 31.12 | 37.62 | 46.43 | 66.33 | 73.87 | 12.60-206.72 |
| Guangyuan | 350 | 40.00±18.11 | 19.92 | | 22.14 | 29.30 | 35.47 | 47.07 | 67.01 | 74.05 | 14.04-206.72 |
| Luzhou | 336 | 38.97±14.12 | 21.67 | | 22.74 | 30.03 | 37.01 | 44.47 | 61.11 | 66.54 | 12.60-150.17 |
| Chengdu | 371 | 42.83±14.09 | 23.24 | | 25.58 | 33.63 | 40.23 | 48.87 | 70.04 | 76.45 | 18.72-136.13 |
| Plateau | 337 | 52.54±19.94 | 20.04 | | 25.00 | 39.49 | 49.38 | 61.74 | 91.70 | 103.58 | 13.59-133.01 |
